# Congenital primary adrenal insufficiency and selective aldosterone defects presenting as salt-wasting in infancy: a single center 10-year experience

**DOI:** 10.1186/s13052-016-0282-3

**Published:** 2016-08-02

**Authors:** Carla Bizzarri, Nicole Olivini, Stefania Pedicelli, Romana Marini, Germana Giannone, Paola Cambiaso, Marco Cappa

**Affiliations:** 1 Unit of Endocrinology and Diabetes, Bambino Gesù Children’s Hospital, Piazza S. Onofrio 4, 00165 Rome, Italy; 2 Department of Chemistry, Bambino Gesù Children’s Hospital, Rome, Italy

**Keywords:** Newborn, Infant, Salt-wasting, Sodium, Aldosterone, Adrenal insufficiency, Pseudohypoaldosteronism

## Abstract

**Background:**

Salt-wasting represents a relatively common cause of emergency admission in infants and may result in life-threatening complications. Neonatal kidneys show low glomerular filtration rate and immaturity of the distal nephron leading to reduced ability to concentrate urine.

**Methods:**

A retrospective chart review was conducted for infants hospitalized in a single Institution from 1^st^ January 2006 to 31^st^ December 2015. The selection criterion was represented by the referral to the Endocrinology Unit for hyponatremia (serum sodium <130 mEq/L) of suspected endocrine origin at admission.

**Results:**

Fifty-one infants were identified. In nine infants (17.6 %) hyponatremia was related to unrecognized chronic gastrointestinal or renal salt losses or reduced sodium intake. In 10 infants (19.6 %) hyponatremia was related to central nervous system diseases. In 19 patients (37.3 %) the final diagnosis was congenital adrenal hyperplasia (CAH). CAH was related to 21-hydroxylase deficiency in 18 patients, and to 3β-Hydroxysteroid dehydrogenase (3βHSD) deficiency in one patient. Thirteen patients (25.5 %) were affected by different non-CAH salt-wasting forms of adrenal origin. Four familial cases of X-linked adrenal hypoplasia *congenita* due to *NROB1* gene mutation were identified. Two unrelated girls showed aldosterone synthase deficiency due to mutation of the *CYP11B2* gene. Two unrelated infants were affected by familial glucocorticoid deficiency due to *MC2R* gene mutations. One girl showed pseudohypoaldosteronism related to mutations of the *SCNN1G* gene encoding for the epithelial sodium channel. Transient pseudohypoaldosteronism was identified in two patients with renal malformations. In two infants the genetic aetiology was not identified.

**Conclusions:**

Emergency management of infants presenting with salt wasting requires correction of water losses and treatment of electrolyte imbalances. Nevertheless, the differential diagnosis may be difficult in emergency settings, and sometimes hospitalized infants presenting with salt-wasting are immediately started on steroid therapy to avoid life-threatening complications, before the correct diagnosis is reached. Physicians involved in the management of infants with salt-wasting of suspected hormonal origin should remember that, whenever practicable, a blood sample for the essential hormonal investigations should be collected before starting steroid therapy, to guide the subsequent diagnostic procedures and in particular to address the analysis of candidate genes.

## Background

Salt-wasting in newborns and infants represents a relatively common cause of emergency admission to hospital and may result in life-threatening complications. The kidneys are responsible of electrolyte homoeostasis, but neonatal kidneys show low glomerular filtration rate and immaturity of the distal nephron leading to reduced ability to concentrate urine. High extra-renal fluid losses often contribute to the increased occurrence of electrolyte disorders. Sodium is the main determinant of serum osmolality, and changes in sodium concentration can result in fluid shifts between intracellular and extracellular compartments. Water influx into the intracellular space swells cells, which can cause cerebral oedema and neurologic sequelae. Newborn and infants with hyponatremia (serum sodium concentration <130 mEq/L) may present with symptoms such as vomiting, irritability, hypotonia and seizures. In the outpatient setting, hyponatremia in infants is very uncommon, but can be caused by excess ingestion of free water and hypotonic fluids, such as overdiluted infant formula or plant milk, or by elevated salt loss with diarrhoea. Most cases of hyponatremia occur in hospitalized infants due to administration of hypotonic fluids, but also occur due to central nervous system pathology, lung disease, or postoperative complications. Severe hyponatremia with or without hyperkalemia, hypochloremia, metabolic acidosis and fasting hypoglycemia is life-threatening in newborns and infants and represents the most typical presentation mode of congenital primary adrenal insufficiency (PAI). The most common form of PAI is the congenital adrenal hyperplasia (CAH) due to 21-hydroxylase deficiency, accounting for more than 75 % of cases [1]. X-linked adrenal hypoplasia congenital (AHC) and familial glucocorticoid deficiency (FGD) represent individually rare monogenic forms of PAI presenting with salt wasting during infancy [1, 2]. Aldosterone synthase deficiency (primary hypoaldosteronism) and aldosterone resistance (pseudohypoaldosteronism: PHA) can mimic clinical presentation of PAI, but glucocorticoid function is always normal in these disorders. However, in many neonatal cases not all the biochemical characteristic are evident at clinical presentation. Hyponatremia is found in 90 % of patients with PAI, while hyperkalemia is seen in only 50 % of patients [1]. Severe salt-wasting with hyponatremia is usually caused by selective mineralocorticoid deficiency, PHA and complete PAI (both glucocorticoid and mineralocorticoid deficiency), but also infants with isolated severe glucocorticoid deficiency (as patients with FGD) and preserved aldosterone secretion may show transient hyponatremia at presentation [1, 2].

### Pseudohypoaldosteronism (PHA)

PHA is characterised by the renal tubular unresponsiveness to aldosterone and presents with hyponatremia, hyperkalaemia and metabolic acidosis with elevated plasma aldosterone and renin concentrations, but normal levels of ACTH and cortisol [3]. The differential diagnosis between congenital PAI and PHA may be challenging in an emergency setting and sometimes newborns and infants presenting with severe hyponatremia, hyperkalaemia, dehydration and metabolic acidosis are immediately started on mineralocorticoid and glucocorticoid therapy to avoid life-threatening complications, before the correct diagnosis is reached [4]. There are two types of primary PHA: PHA1 is characterized by failure to thrive, anorexia, nausea, and vomiting with hypotension, hyperkalemia, hyponatremia and acidosis, accompanied by high aldosterone levels; in contrast, PHA2 or type four renal tubular acidosis is due to the failure of the kidney to appropriately secrete potassium. Individuals with PHA2 show early-onset hypertension readily responsive to sodium restriction and thiazide diuretics, hyperkalemia, hyperchloremic acidosis, normal or low plasma renin activity, and subnormal plasma aldosterone level relative to the potassium level [5]. PHA1 is further categorized into two forms: autosomal recessive PHA1 due to a defect in the genes *SCNN1A*, *SCNN1B* and *SCNN1G* encoding for the subunits α, β and γ of the epithelial sodium channel (ENaC), respectively; and autosomal dominant PHA1 due to mutations of the *NR3C2* gene encoding for the mineralocorticoid receptor (MR) [5, 6]. Patients with autosomal recessive PHA1 present not only severe lifelong renal salt-wasting, but also salt-wasting from the lung, colon, sweat and salivary glands. As a result, these patients experience recurrent salt-wasting crises, pulmonary infections, congestion, coughing and wheezing, cholelithiasis, skin infections, and miliaria rubra. Initial presentation is usually severe dehydration early after birth, but sometimes autosomal recessive PHA1 has been diagnosed by neonatal cutaneous symptoms. These individuals are prone to life-threatening salt-losing crises throughout their lives. The pulmonary symptoms, clinically similar to those of cystic fibrosis, are due to poor absorption of liquids from airway surfaces and occur within weeks or months from birth. To note, there have been no described cases of neonatal respiratory distress in these patients. Instead, they experience recurrent lung infections such as bronchopneumonia by different pathogens as pseudomonas, pneumococcus, Staphylococcus aureus, Klebsiella, and Serratia [7]. Autosomal dominant PHA1 clinical expression is milder and confined to the kidney. Patients with autosomal dominant PHA1 generally present with renal salt wasting that subsides after early childhood. However, the presentation varies widely; it can occur in infancy with salt wasting, growth retardation, failure to thrive and occasionally death; or it can present in adulthood. It may be unmasked by an illness that impairs oral intake of fluids and salt or results in additional losses, such as viral gastroenteritis.

Secondary (transient) PHA is confined to the kidneys and has been described in infants and children with obstructive uropathy, pyelonephritis, tubulointerstitial nephritis, sickle cell nephropathy, systemic lupus erythematosus. Secondary PHA has been also related to drugs like non-steroidal anti-inflammatory drugs and potassium sparing diuretics [3, 6]. It represents the result of a transient aldosterone resistance related to the kidney infection, in children with or without a predisposing renal malformation. The prognosis is good and the PHA usually reverts with the resolution of the infection.

### Primary selective hypoaldosteonism

Primary selective hypoaldosteonism is a rare autosomal recessive disorder causing salt-wasting, accompanied by vomiting, failure to thrive, and dehydration during the first months of life. It is due to mutations in the *CYP11B2* gene encoding for a cytochrome P450 enzyme, termed aldosterone synthase (CYP11B2, P450c11AS). This enzyme catalyzes the terminal steps in aldosterone biosynthesis comprising the conversion of 11-deoxycorticosterone to corticosterone, 18-hydroxycorticosterone, and finally aldosterone. The aldosterone synthase is expressed exclusively in the cells of the adrenal zona glomerulosa, limiting the synthesis of aldosterone to that zone [8].

### Familial glucocorticoid deficiency (FGD)

FGD, also known as hereditary unresponsiveness to ACTH, is characterized by lack of response to ACTH leading to adrenal insufficiency with subnormal glucocorticoid levels and remarkably elevated plasma ACTH levels. Aldosterone secretion is generally preserved, but hyponatremia at clinical presentation is common in infants [9]. Mutations in the ACTH receptor gene (*MC2R*) account for 25 % of FGD cases. Mutations in the melanocortin receptor accessory protein gene (*MRAP*) account for 20 %, and mutations in the steroidogenic acute regulatory protein (STAR) gene account for 2.5 % of cases. Recently, mutations in the mini-chromosome maintenance-deficient four homologue (*MCM4*) and nicotinamide nucleotide transhydrogenase (*NNT*) genes, involved in DNA replication and antioxidant defense, and mutations in *TXNRD2* gene, encoding the mitochondrial selenoprotein thioredoxin reductase 2, have been also implicated [10–12].

### X-linked adrenal hypoplasia * congenita* (AHC)

X-linked adrenal hypoplasia *congenita* (AHC) is a potentially life-threatening condition characterized by primary adrenal insufficiency associated with hypogonadotropic hypogonadism Incidence was estimated to be about 1/12.500 births. This genetic disorder is caused by mutations or deletions in *NR0B*1 gene located on the short arm of chromosome X.* NR0B1* gene encodes for a nuclear receptor protein (DAX-1) which plays a key role in regulating the development of adrenal cortex, gonads, hypothalamus and pituitary gland. Salt-wasting crisis is often the first clinical manifestation of AHC occurring during the neonatal period, but timing and clinical presentations of the disease may be variable and characterized by non specific symptoms which can occur during childhood or adolescence [13].

### Autosomal adrenal hypoplasia *congenita*

The first description of steroidogenic factor 1 (SF-1) protein abnormalities as a cause of human adrenal and testicular dysgenesis was published more than 15 years ago, and the spectrum of SF-1 associated conditions has expanded rapidly. SF-1 is encoded by the *NR5A1* gene. Isolated PAI has been rarely associated with disruption of SF-1, whereas a range of reproductive phenotypes are more commonly seen, including 46 XY disorders of sex development (DSD), with or without associated PAI, male infertility and premature ovarian failure. Most disease-associated variants are heterozygous, with rare homozygous changes described [13].

## Methods

**Fig. 1:**
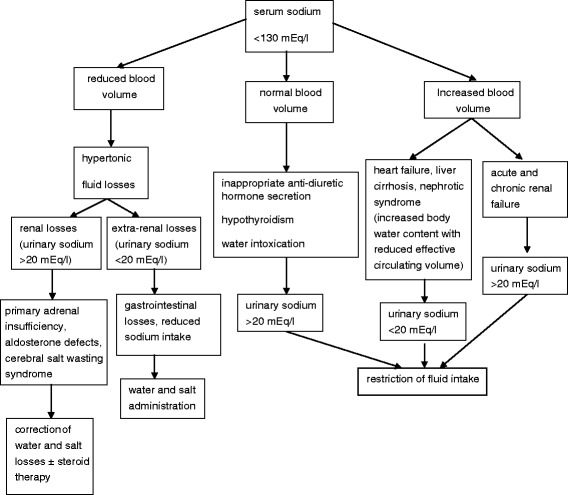
Differential diagnosis and treatment of hyponatremia in infancy

The study was conducted at Bambino Gesù Children’s Hospital (Rome, Italy). A retrospective chart review was conducted for all patients with hyponatremia hospitalized during the first year of life, from 1^st^ January 2006 to 31^st^ December 2015. The selection criterion was represented by the referral to the Endocrinology Unit for hyponatremia (serum sodium <130 mEq/L) of suspected endocrine origin at admission. The diagnostic algorithm routinely used in our hospital to address the differential diagnosis of hyponatremia in infancy is summarized in Fig. 1. The following data were collected from the charts: clinical, biochemical and hormonal features at initial presentation, additional clinical features, and results of mutation analysis. The different candidate genes were sequenced to identify mutations, based on clinical characteristics and hormonal results of individual patients.

### Hormone assays

Plasma ACTH and serum cortisol were measured by immunoluminometric assay and luminescence immunoassay (Nichols Institute, San Juan Capistrano, CA), respectively. Mean inter- and intrassay variability coefficients of variation were <10 % and <4.8 %, respectively, for both methods. Commercial kits were used to estimate plasma renin (RIA, Sanofi-Pasteur), with mean intra- and interassay coefficients of variation 7, 9 % and 10 %, respectively.

## Results

**Fig. 2:**
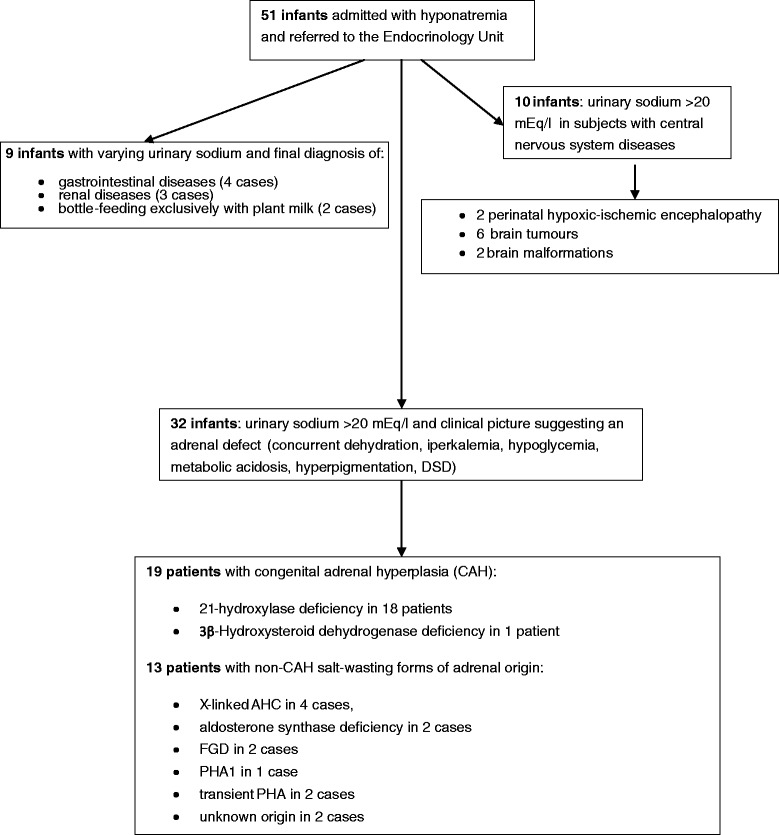
Flow diagram summarizing the recruitment procedure and the results. (CAH: congenital adrenal hyperplasia; AHC: X-linked adrenal hypoplasia congenital, FGD: familial glucocorticoid deficiency, PHA: pseudohypoaldosteronism, DSD: disorders of sex development)

A total of 51 infants, admitted with significant hyponatremia (<130 mEq/l) and referred to the Endocrinology Unit were identified (Fig. 2).

At the end of the diagnostic work-up, hyponatremia was identified as related to chronic unrecognized salt losses or reduced sodium intake in nine infants (17.6 %): four infants with gastrointestinal diseases; three infants with renal diseases; two cases of bottle-feeding exclusively with over diluted rice milk not enriched with vitamins or minerals. In 10 infants (19.6 %) hyponatremia was related to central nervous system diseases, suggesting syndrome of inappropriate anti-diuretic hormone secretion and/or cerebral salt wasting syndrome. All these patients were excluded from the study.

In 19 patients (37.3 %) the final diagnosis was CAH. A defect of 21-hydroxylase (*CYP21A2* gene) was identified in 18 CAH patients (five females). One newborn male with perineal hypospadia and microphallus was found affected by CAH related to 3β-Hydroxysteroid dehydrogenase (3βHSD) deficiency due to mutations in *HSD3B2* gene. Despite all the five newborn girls with 21-hydroxylase CAH showed severe virilization of the external genitalia (one girl with Prader stage 5; four girls with Prader stage 4), they had been discharged from the peripheral hospital where they were born with the alert about the possibility of a salt-wasting crisis and an appointment for an endocrinologic evaluation in the outpatient clinic, without undergoing any first-line hormonal investigations before discharge.

Thirteen patients (25.5 %) were found affected by non-CAH salt-wasting forms of adrenal origin (Table 1). Four familial cases carrying the same *NROB1* (DAX1) mutation were identified in two families (four boys born from two sisters). Two unrelated girls were found affected by aldosterone synthase deficiency due to mutation of the *CYP11B2* gene. Two unrelated children (one male) showed FGD due to *MC2R* mutations. One girl showed autosomal recessive PHA1 related to compound heterozygosity for two variants in intron eight of the *SCNN1G* gene (c.1294 + 5G > A e c.1295-10 T > A). In this case bioinformatics analysis showed that the first variation abolished the 5' splice site and was probably pathogenic, while the second variation was predicted to abolish the 3' splice site and to introduce a cryptic splice site of unknown significance. Two cases of transient PHA were identified, both of them were related to urinary infections in patients with renal malformations. Finally, in two infants (one male) with a clinical and hormonal diagnosis of PAI, the final genetic aetiology was not identified. Based on individual clinical details,* CYP21A2*, *CYP11B1*,* HSD3B2*, *CYP17A1*,* POR*, *NROB1* (DAX1) and *NR5A1* (SF1) genes were sequenced in both these children. Very long chain fatty acids (VLCFA) were measured in both patients, and were in the normal range. In the boy, Smith-Lemli-Opitz syndrome was also excluded by the study of *DHCR7* gene, encoding for 7-dehydrocholesterol reductase.

**Table 1:** Characteristics of infants with non-CAH salt-wasting forms of adrenal origin

	Age at onset (days)	Sex	Ethnicity	Ancestry	Clinical presentation	Additional clinical features	pH	Nammol/L(135 –145)	K mmol/L(3.5 – 5.5)	Cl mmol/L(95–110)	Glucose mg/dl (50–110)	Blood pressure mmHg	ACTH pg/ml (5–55)	Cortisol mcg/dl (5–20)	Aldosterone pg/ml (50–900)	Renin pg/ml (5–270)	Gene	Mutation
1	21	M	Caucasian	Romanian	Salt wasting crisis, fever, fatigue, poor feeding	NR		130	5.74	92	89	72/36	286	5.6	165	784	*NROB1*	P353LfsX387
2	21	M	Caucasian	Romanian	Poor feeding, dehydration, hyperpigmentation	NR		127.2	5.9	95	48	79/40	356	6.5	56	668	*NROB1*	P353LfsX387
3	3	M	Caucasian	Romanian	Poor feeding	NR		133	5.4	99	62		184	4.8	54	452	*NROB1*	P353LfsX387
4	8	M	Caucasian	Romanian	Poor feeding, recurrent vomiting	NR	7.27	127	6.91	99	66		176	14		528	*NROB1*	P353LfsX387
5	18		Caucasian	Italian	Dehydration, failure to thrive, poor feeding	NR	7.12	127	9.53	93	57	65/38	59.3	22.4	>800	639	*SCNN1G*	c.1294+5G>A, c.1295-10T>A
6	60	F	Caucasian	Italian	failure to thrive, recurrent vomiting	NR	7.43	124	6.1	94	68	73/40	19.1	14.9	98	826	*CYP11B2*	Q170X, E198D, V386A
7	24	F	Caucasian	Italian	failure to thrive, recurrent vomiting	NR	7.38	120	6.72	99	69	72/35	15.8	5.7	255	1250	*CYP11B2*	p.T185I, p.E198D, p.V386A
8	1	M	Caucasian	Italian	Hypoglycemia, respiratory distress, hyperpigmentation	cholestatic jaundice	7.27	126	3.77	97	50	70/35	>1250	<0.2	224	107	*MC2R*	T159K, A233D
9	1	F	Caucasian	Italian	Hypoglycemia, poor feeding, recurrent vomiting, hyperpigmentaiton	cholestatic jaundice	7.29	128	3.92	98	24	69/34	1248	0.49	181	181	*MC2R*	T159K
10	28	M	Caucasian	Albanian	poor feeding, recurrent vomiting, fever	Urinary sepsis, right kidney hypoplasia	7.30	129	7.56	97	52	80/40	23	13.1	933	456	Transient PHA	
11	90	M	Caucasian	Romanian	poor feeding, hypotonia, recurrent vomiting, fever	Urinary sepsis, right renal pelvis distension	7.31	122	7.34	96	53	80/43	43	12	989	563	Transient PHA	
12	180	F	Caucasian	Italian	poor feeding, recurrent vomiting, diarrhea	Intrauterine growth retardation	7.22	110	6.3	74	44	88/44	>1250	2.91	155	677	unknown	
13	15	M	Caucasian	Italian	Dehydration, failure to thrive, recurrent vomiting, hyperpigmentation	46 XY,DSD, dysmorphic features, retinopathy, developmental delay	7.23	122	6.9	89	43		>1250	2.3	98	478	unknown	

## Discussion

PAI is uncommon in the Western population; 90 to 140 subjects per 1 million people have been estimated to be affected [14]. In contrast to the predominance of autoimmune adrenal insufficiency in adults, most cases of PAI in childhood show an inherited, monogenic origin [15]. Genetic causes of paediatric PAI can be classified into four major groups according to the underlying pathogenesis: impaired steroidogenesis, adrenal hypoplasia, familial glucocorticoid deficiency (FGD), and adrenal destruction. Congenital adrenal hyperplasia (*CYP21A2, CYP11B1, HSD3B2, CYP17A1, POR* defects) constitutes the largest subgroup of impaired steroidogenesis and represents the most common cause of PAI in children [15]. In contrast, different individually rare causes of PAI as X-linked AHC (*NR0B1*/DAX-1), autosomal adrenal hypoplasia *(NR5A1*/SF-1), congenital lipoid adrenal hyperplasia (*CYP11A1*, *STAR* gene defects), FGD, adrenal autoimmune destruction and adrenoleukodystrophy (*ABCD1*) are now well established [15, 16].

Perry et al. [16] reported their 20-year experience with PAI in 103 children <18 years old in Montreal, Canada. An aetiology of PAI was identified in 94 % of cases: 72 % were affected by CAH, 13 % had autoimmune adrenal insufficiency, and the remaining 15 % had adrenoleukodystrophy, rare syndromes (Wolman, Zellweger, etc.), or unexplained adrenal insufficiency.

In a study performed in Melbourne, Australia [17] in which only non-CAH cases were reported, there were five cases each of autoimmune adrenal insufficiency, adrenoleukodystrophy, and X-linked AHC and one case of the IMAGE syndrome (characterized by intrauterine growth retardation, metaphyseal dysplasia, adrenal hypoplasia congenita, and genital anomalies).

A recent study performed in a population from the island portion of Newfoundland and Labrador, Canada, reported mutation analysis of candidate genes in 11 patients with PAI. The aetiology of PAI was identified in nine patients. One had a homozygous *MC2R* mutation associated with FGD. Two had the same homozygous mutation in the autoimmune regulator (*AIRE*) gene, which is associated with type 1 autoimmune polyglandular syndrome. One patient had a heterozygous change in *AIRE* gene of undetermined significance. Five were homozygous for the same mutation of the gene encoding the steroidogenic acute regulatory (STAR) protein causing non-classic lipoid CAH. In the remaining two patients, no clear aetiology was identified. In this study, the high proportion of patients with non-classic lipoid CAH strongly suggests a founder effect in a relatively isolated population [18].

It is now emerging that a considerable overlap exists in the clinical and biochemical presentation of the different genetic forms of PAI. FGD can present with salt loss suggestive of adrenal hypoplasia, and alterations in STAR and CYP11A1 resulting in partial loss of protein function may have a predominant FGD-like phenotype, with glucocorticoid deficiency without genital abnormalities in 46,XY newborns [18, 19].

To our knowledge, this is the first study focusing on salt-wasting and hyponatremia as clinical presentation of congenital adrenal defects in infancy. Our Institution is the main Children’s Hospital of Lazio region, in the central Italy. In Lazio region, the neonatal screening for CAH is not performed. Our Institution does not include a department of Obstetrics and sick newborn babies are referred to us from peripheral birth hospitals. In female babies, CAH is more easily suspected due to the presence of genital abnormalities. Female babies are usually referred immediately after birth, before the onset of an overt electrolyte imbalance. This probably explains why in our population, selected on the basis of hyponatremia at the first observation, the number of females with CAH was significantly lower than the number of males. Nevertheless, all the five girls with CAH showed hyponatremia at the first outpatient consultation, without overt dehydration and salt wasting, and were admitted to confirm CAH diagnosis and start treatment. As expected, the number of patients affected by the other forms of salt wasting of adrenal origin was low, and a genetic cause was not found in two patients. Surprisingly, in our study the proportion of patients with primary selective hypoaldosteronism (two unrelated cases) was relatively high, considering that the estimated incidence of this defect is probably <1/1,000,000/year [20, 21]. One case of aldosterone synthase (CYP11B2) deficiency and the case affected by 3β-Hydroxysteroid dehydrogenase (3βHSD) deficiency were previously described [22, 23].

Severe salt-wasting with hyponatremia is caused by mineralocorticoid deficiency or complete adrenal insufficiency (both glucocorticoid and mineralocorticoid deficiency), but also infants with isolated severe glucocorticoid deficiency and preserved aldosterone secretion may present with mild hyponatremia, which is usually transient. Interestingly, both patients with severe FGD due to *MC2R* mutations showed mild hyponatremia at onset, without dehydration and overt salt-wasting. It has been described that in severe glucocorticoid deficiency plasma anti-diuretic hormone (ADH) levels are elevated, because of the decreased effective circulating blood volume, impairing the ability to dilute the urine. Cortisol acts as a negative feedback mechanism on both CRH and ADH secretion. ADH is cosecreted with CRF by the paraventricular nuclei. If cortisol levels are chronically low, the amount of ADH can continue to increase causing dilutional hyponatremia [2].

## Conclusions

Emergency management of newborns infants presenting with dehydration and salt wasting requires correction of water losses and treatment of electrolyte imbalances. Nevertheless, the differential diagnosis between congenital PAI and PHA may be difficult in emergency settings, and sometimes hospitalized infants presenting with salt-wasting and metabolic acidosis are immediately started on mineralocorticoid and glucocorticoid therapy to avoid life-threatening complications, before the correct diagnosis is reached [4, 24, 25]. Steroid therapy (with both glucocorticoids and mineralocorticoids) is life-saving in PAI, while it is not effective in restoring salt and water balance in PHA. After correction of dehydration, children with aldosterone synthase deficiency require mineralocorticoid therapy only, even if stress doses of glucocorticoids administered intravenously are able to restore the electrolyte balance because cortisol is able to bind the MR [20, 21]. Physicians involved in the management of infants with salt wasting of suspected endocrine origin should remember that, whenever practicable, a blood sample for the essential hormonal investigations (ACTH, cortisol, 17 OH progesterone, androstenedione, renin, aldosterone) should be collected before starting steroid therapy, to guide the subsequent diagnostic procedures and in particular to address the analysis of candidate genes.

## Abbreviations

ADH, anti-diuretic hormone; AHC, adrenal hypoplasia *congenita*; CAH, congenital adrenal hyperplasia; DSD, disorders of sex development; ENaC, epithelial sodium channel; FGD, familial glucocorticoid deficiency; MR, mineralocorticoid receptor; PAI, primary adrenal insufficiency; PHA, pseudohypoaldosteronism
